# Characterization and Antimicrobial Susceptibility of Pathogens Associated with Periodontal Abscess

**DOI:** 10.3390/antibiotics9100654

**Published:** 2020-09-29

**Authors:** Muhammad Irshad, Mohammad Khursheed Alam, Ahmad Alawneh, Mohammed Abdullah Alhadi, Ahmed Abdullah Alhadi, Yasser Saleh Almunajem, Fesal Farag Alanezi, Sharafi Abdullah Al Sagoor, Abdulrahman Mudaysh Bajawi, Ahmed Ali Alfawzan, Mohammad Amjad Kamal

**Affiliations:** 1 Department of Oral Pathology, Rehman College of Dentistry, RMI, Hayatabad Phase V, Peshawar, KP, Peshawar 25000, Pakistan; 2 College of Dentistry, Jouf University, Sakaka 72345, Saudi Arabia; dralam@gmail.com; 3 Dental Department, Jordanian Royal Medical Services, Amman 11180, Jordan; ahmadalawnehdentist@yahoo.com; 4 Aljouf Specialist Dental Center, MOH, Sakaka 72345, Saudi Arabia; dr.m.alhadi@gmail.com; 5 Ministry of Health in Saudi Arabia, Riyadh 11176, Jurisdiction; dr.alhadi17@gmail.com (A.A.A.); dr.almunajem@gmail.com (Y.S.A.); dr.faisaldental@gmail.com (F.F.A.); shanals1988@gmail.com (S.A.A.S.); albajawi15@gmail.com (A.M.B.); 6 Department of Preventive Dentistry, College of Dentistry in Ar Rass, Qassim University, Ar Rass 51921, Saudi Arabia; dr.ahmed.alfawzan@qudent.org; 7 King Fahd Medical Research Center, King Abdulaziz University, Jeddah 21589, Saudi Arabia; prof.ma.kamal@gmail.com; 8 Enzymoics, 7 Peterlee Place, Hebersham, NSW 2770, Australia; 9 Novel Global Community Educational Foundation, Hebersham, NSW 2770, Australia

**Keywords:** periodontitis, antimicrobial resistance, periodontal abscess, amoxycillin, metronidazole, tetracycline, azithromycin

## Abstract

Knowledge of microbial composition and antimicrobials’ susceptibility to periodontal abscesses is vital for their successful treatment. The current study aims to provide a thorough overview of the clinical and microbial features of periodontal abscesses of the local community. The study was carried out at Rehman College of Dentistry, Peshawar, Pakistan between December 2019 to March 2020. Clinical measurements and microbial samples were collected from 45 subjects. Microbial samples were anaerobically cultured for the growth of selected bacterial species. E-test was used to assess the susceptibility of bacterial species grown from the patient samples to amoxicillin, azithromycin, metronidazole, and tetracycline. The majority of affected patients had preexisting chronic periodontitis. All abscesses clinically demonstrated bleeding on probing and suppuration. The periodontal abscess was most commonly associated with lower incisors and canines, followed by lower molars and then upper incisor and canine teeth. *Fusobacterium* spp. (73%) was the most frequently detected species followed by *Prevotella intermedia/nigrescens* (65%), *Porphyromonas gingivalis* (46%) and *Aggregatibacter actinomycetemcomitans* (24%). The detected clinical isolates of certain bacteria demonstrated resistance to all tested antibiotics except azithromycin. We conclude that *Fusobacterium* spp., *P. intermedia/nigrescens*, *P. gingivalis, C. rectus, T. forsythia* and *A. actinomycetemcomitans* are closely associated with periodontal abscess. Bacterial species associated with periodontal abscess demonstrated some level of antimicrobial resistance to amoxicillin, metronidazole and tetracycline while antibiotic resistance to azithromycin could not be demonstrated.

## 1. Introduction

A localized, pus-forming infection of the surrounding tissues of a periodontal pocket is called a periodontal abscess [1]. The periodontal tissues appear edematous accompanied by bleeding on probing (BOP), suppuration and periodontal pocket formation [2]. Pre-existing chronic periodontitis has been identified as one of the main risk factors for periodontal abscess [2]. Trauma to periodontal tissues is another major predisposing factor in patients without chronic periodontal disease [3]. Presence and recurrence of periodontal abscess has been associated with tooth loss in chronic periodontitis patients [4] which highlights the importance of early treatment of acute periodontal abscess.

The microbiota associated with periodontal abscess shows a close resemblance to that of chronic periodontitis [5]. Hence, the development of periodontal abscess indicates a shift in the local microenvironment of the periodontal pocket which results in the growth of periodontopathic microorganisms and abscess formation. Bacterial species most commonly associated with periodontal abscess include *Fusobacterium nucleatum, Prevotella intermedia, Porphyromonas gingivalis*, *Actinobacillus actinomycetemcomitans* and *Comphylobacter rectus* [5,6].

Treatment of periodontal abscess depends upon the severity of damage to the periodontium. Generally, surgical drainage of the periodontal abscess through the pocket is followed by debridement and irrigation with saline solution. In more severe cases, tooth extraction might be the only option. In addition, adjunctive antibiotic therapy is sometimes indicated. The recommended antibiotics include tetracycline, penicillin, metronidazole, amoxicillin/clavulanate and azithromycin [7,8,9]. Azithromycin is a macrolide antibiotic which has been found effective in the treatment of periodontitis [10]. Azithromycin is not a first-line antibiotic for the treatment of oral infections; however, it can be used in patients allergic to amoxicillin. Most previous studies on microbiology of periodontal abscess have focused on the use of conventional antibiotics such as amoxicillin and metronidazole. The effectiveness of azithromycin against bacteria associated with periodontal abscess has never been reported previously.

Knowledge of the most common pathogens implicated in periodontal abscess and their susceptibility profiles is necessary for a rational antibiotic prescription. Microbial composition and antimicrobial resistance of bacteria associated with periodontal abscess vary in different populations [5,6]. To the best of our knowledge, there is no study from Pakistan reporting on the characterization and antibiotic susceptibility profiles of bacterial species associated with periodontal abscess. Therefore, this study aimed to evaluate the clinical and microbiological characteristics of periodontal abscess and determine the antimicrobial susceptibility profiles of bacterial species closely associated with periodontal abscess.

## 2. Materials and Methods

Informed consent was obtained from all patients participating in the study and the study was approved from the ethics committee of the institution (EC-Ref; RCD-20-05-014). Forty-five patients with at least one periodontal abscess, attending the oral surgery department of Rehman College of Dentistry between December 2019 and March 2020 were included in the study. A periodontal abscess was clinically diagnosed based on the following criteria;

1. Probing pocket depth ≥ 6mm
2. Gingival swelling with suppuration (spontaneous or provoked)
3. Absence of periapical pathology on a periapical radiograph

Patients with pulpitis/pulp necrosis, use of systemic antibiotics in the 3 months before the examination and uncontrolled diabetes were excluded from the study.

Clinical examination

Periodontal charting was done for all patients. The recorded clinical parameters include BOP, pain, redness, suppuration, periodontal probing depth (PD) with a standardized periodontal probe (Hawe Click-Probe, Hawe Neos Dental, Switzerland). Tooth mobility was evaluated according to the following scoring criteria:

1—horizontal displacement of 1 mm,
2—horizontal displacement >1 mm and
3—horizontal and vertical displacement >1 mm.

Periapical radiographs were used to assess bone loss and categorized into mild (1/3rd of root length), moderate (2/3rd of root length) and severe (>2/3rd of root length)

### 2.1. Microbiological Analysis

A sterile gauze was used to remove the supra-gingival plaque and the tooth was isolated with cotton rolls before taking the subgingival sample. Subgingival samples were collected by inserting 3 sterile paper points into the deepest part of the periodontal pocket for 15 s. Subsequently, paperpoints were transferred to 5 ml sterile tubes with reduced transport fluid. Plaque samples were cultured for bacterial growth under anaerobic conditions as described elsewhere [11]. Subgingival plaque samples were serially diluted and cultured on 5% horse blood agar (HBA, Oxoid no.2, Basingstoke, UK) supplemented with hemin (5 mg/L) and menadione (1 mg/L). Trypticase soy-serumbacitracin-vancomycin (TSBV) plates were used as a culture medium for the *A. actinomycetemcomitans* growth. After incubating blood agar plates under anaerobic conditions (80%N_2_, 10%H_2_, and at 10%CO_2_) at 37 °C and TSBV plates in air with 5%CO_2_ for up to 15 days, the plates were examined for the presence of bacterial colonies. The presence and proportions of *Fusobacterium nucleatum, Prevotella intermedia, Porphyromonas gingivalis*, *Tannerella forsythia*, *Comphylobacter rectus*, *Actinobacillus actinomycetemcomitans* and Gram-negative enteric rods were recorded. Bacterial colonies were counted and expressed as colony-forming units per ml (CFU/mL). Colony morphology, Gram staining and microscopy, anaerobic growth, fermentation of glucose and indole were used to identify bacterial species.

### 2.2. Antimicrobial Susceptibility

Standard E-test^®^ (bioMérieux (Marcy-l’Etoile, France) was used to test the antimicrobial susceptibility of *P. gingivalis*, *A. actinomycetemcomitans* and *P. intermedia/nigrescens* to amoxicillin, azithromycin, tetracycline and metronidazole. Colonies of the selected bacterial species were suspended in normal saline solution and colony counts were adjusted to 3 × 10^8^ CFU/mL (MacFarland 1.0 standard). A standard bacterial inoculum (0.1 mL) was plated on 5% HBA supplemented with hemin (5 mg/L) and menadione (1 mg/L). E-test strips were carefully positioned onto the HBA surface and anaerobically incubated for 4 days. The plates were examined after 96 h of incubation under anaerobic conditions. Minimal inhibitory concentration (MIC) was defined as the reading at the juncture of the bacterial zone of inhibition and the E-strip. The cut-off points used for the antimicrobials were as described earlier [12,13,14,15] (amoxicillin, azithromycin and tetracycline ≤4 μg/mL, for metronidazole ≤8 μg/mL). All the laboratory work was carried out at the microbiology laboratory of Veterinary Research Institute, Peshawar, Pakistan.

The sample size was calculated using G*Power software, version 3.1.9.4. The calculated sample size was 56 at an effect size of 0.482, alpha of 0.05 and study power of 0.80.

Data were organized using Microsoft Excel 2016 and data analysis including the determination of frequencies, percentages and graphs were carried out using GraphPad Prism software (version 7.00 for Windows, San Diego, CA, USA).

## 3. Results

Table 1 presents data from 45 patients (56 periodontal abscesses) with a mean age of 45.4 ± 7.5 years.

Clinical parameters of periodontal abscess are presented in Table 2. All abscesses presented with BOP, 89% of lesions had redness while 96% of cases presented with suppuration. The majority of the cases showed increased probing pocket depth (8.5 ± 2.5 mm), evidence of bone loss on periapical radiographs and tooth mobility. Most affected teeth showed grade 1 and 2 mobility while the radiographic bone loss was moderate to severe in most cases. History of current or past periodontitis was found to be a strong predictor of the development of periodontal abscess and accounted for approximately 67% of cases. Periodontal therapy accounted for about 3.5% abscesses.

**Figure 1:**
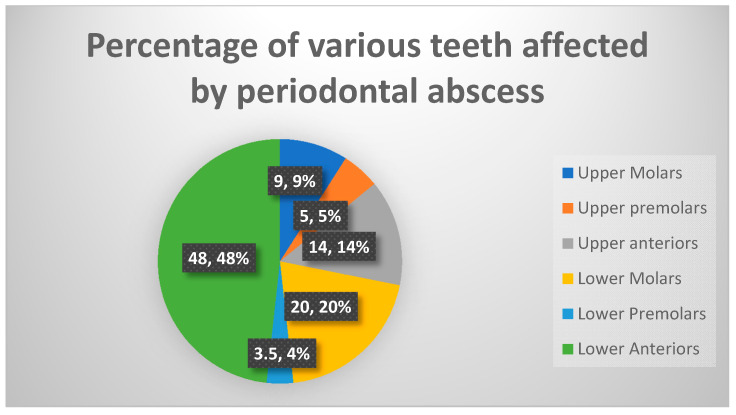
Frequency distribution (%) of periodontal abscess according to the affected teeth.

Figure 1 presents the frequency of periodontal abscess according to the affected teeth. Lower incisor and canine teeth were most affected (48.5%) as a group (*p* < 0.001), followed by lower molars (20%) and upper anterior teeth (14%). Premolars (upper and lower) were the least commonly affected teeth by periodontal abscess (3.5 and 5.5%, respectively). Upper molars accounted for approximately 10% of periodontal abscess cases in our study. The periodontal abscess should always be ruled out in diagnosing pathologies associated with lower incisors and canines since these are the most commonly affected teeth by a periodontal abscess in our population.

Table 3 presents the frequency of detection and loads of the bacterial species obtained from subgingival plaque samples of periodontal abscesses. *Fusobacterium* spp. were the most frequently detected (73%), *p* = 0.013 followed by *P. intermedia/nigrescens*, (64%). *P. gingivalis* was detected in 46% while *T. forsythia* in 13% of the abscesses. *A. actinomycetemcomitans* was recovered from 25% of cases. *Campylobacter* spp. was detected in 6 out of 56 samples (11%). We also detected Gram-negative enteric rods in 11 cases (20%). The percentage of *P. intermedia/nigrescens* was the highest (10.35 ± 15.40, *p* = 0.032) while that of. *A. actinomycetemcomitans* was the lowest (0.11 ± 0.23) among the cultivable bacterial species.

Table 4 presents the susceptibility of selected bacterial isolates to commonly prescribed antimicrobials. Out of 6 tested *A. actinomycetemcomitans* isolates, 1, 4 and 2 isolates displayed resistance to Tetracycline, Metronidazole and Amoxicillin, respectively. Out of 10 isolates of *P. gingivalis* tested only one showed resistance to Metronidazole. Two out of 10 isolates of *P. intermedia/nigrescens* showed resistance to amoxicillin. Azithromycin was effective against all the bacterial isolates tested. Among the studied bacterial species, a higher frequency of antimicrobial resistance was observed in *A. actinomycetemcomitans* isolates compared to either *P. gingivalis* or *P. intermedia/nigrescens*, regardless of the antimicrobials used.

Data presented here gives us an overview of the most commonly isolated bacterial species, their relative loads and susceptibility to commonly prescribed antibacterial agents. Therefore, it is imperative to consider these results when prescribing adjunctive antibacterial agents to patients with periodontal abscess.

## 4. Discussion

In the current study, most cases of periodontal abscess were found to be associated with pre-existing periodontitis, thus, confirming findings of other studies where periodontal abscesses are considered as a complication of chronic periodontitis [4,5]. We also found that some cases of periodontal abscess can be attributed directly to periodontal therapy. Periodontal instrumentation could be responsible for the closure of periodontal pockets and thus lead to abscess formation.

In this study, lower incisors were found to be the most commonly affected teeth. Our findings agree with the results of another study [16] with slightly larger sample size. However, in another study molar teeth were reported to be the most commonly affected by periodontal abscess [5].

Close similarities have been reported in the microbiota associated with periodontal abscess and periodontitis. *Fusobacterium* spp., *P. intermedia/nigrescens* and *P. gingivalis* were the most frequently detected bacteria in the current study which is in line with some previous studies [6]. Therefore, these bacteria seem to have a close association with periodontal abscess. In contrast to an earlier report [5], where *P. gingivalis* was found to have the highest relative proportions, we detected the highest relative proportions of *P. intermedia/nigrescens* (10.35 ± 15.40) out of all bacteria studied. In one report [5], the prevalence of *T. forsythia* was found (66.7%) to be much higher than in the present study (15%). Differences in demographics or incubation times could explain their lower frequency. Differences in the prevalence of *A. actinomycetemcomitans* have also been found in various studies and have been reported ranging from 0 to 30% [6,7]. We found *Campylobacter rectus* in 11% of cases which is in contrast to Herrera et al. [5] and Häfstrom et al. [7] who reported different prevalence values (4.2% and 80%, respectively). These variations can be partly attributed to the different demographic parameters of the studied populations in these studies. Enteric Gram-negative rods have previously been proposed as possible superinfecting agents in periodontal diseases [17,18] however, their presence in periodontal abscesses is intriguing. As enteric Gram-negative rods have important virulence factors that play an important role in tissue invasion [19,20], we suggest a potential role of these bacteria in the rapidly progressing tissue breakdown witnessed in periodontal abscess. In addition, the association of some previously unrelated microbes such as *D. pneumosintes,* with periodontal abscess has been reported in recent studies [21,22].

The results of our study substantiate findings of previous reports which demonstrate the resistance of *P. intermedia* and *P. nigrescens* to amoxicillin, metronidazole and tetracycline [13,14]. In the current study, none of the studied bacteria demonstrated resistance to azithromycin. We know from previous studies that the emergence of antibiotic resistance is highly dependent on the level of antibiotics consumption in a region [23,24,25]. Therefore, the emergence of resistant strains to amoxicillin, metronidazole and tetracycline could be explained by the higher frequency of their prescription by clinicians and over-the-counter availability in the region. In contrast, azithromycin is not as frequently prescribed for the treatment of dental and medical infections, therefore, it is still effective against oral bacteria.

Our antimicrobial susceptibility results should be carefully extrapolated to other regions/populations. Moreover, these laboratory findings must be substantiated by large-scale clinical trials for clinical correlation and prescription guidelines.

Efforts on individual and policy levels should be focused to discourage the indiscriminate use of antimicrobials which can result in the emergence of resistant bacterial strains. Unnecessary use of antimicrobials must be avoided by all medical professionals and prescription must be based on culture/sensitivity testing rather than their empirical use.

## 5. Conclusions

The most significant factor for the development of periodontal abscess is untreated chronic periodontitis. *Fusobacterium* spp., *P. intermedia/nigrescens*, *P. gingivalis, C. rectus, T. forsythia* and *A. actinomycetemcomitans* show a close association with periodontal abscess. Bacterial species associated with periodontal abscess demonstrated some degree of antimicrobial resistance to amoxicillin, metronidazole and tetracycline although none of the studied bacteria showed resistance to azithromycin.

## Figures and Tables

**Table 1:** Demographic characteristics of studied population

Patient Demographics	Frequency, *n*
Subjects	45
Gender	*F* = 22, M = 23
Age, mean ± SD	45.4 (±15.5) years
Current smoker, *n* (%)	6 (11%)

**Table 2:** Frequencies of various clinical parameters of periodontal abscess.

Clinical Parameter	Frequency (*n* = 56), *n* (%)
BOP	56 (100)
PD (mm ± SD)	8.5 ± 2.5
Redness	50 (89)
Suppuration	54 (96)
Mobility	
1	20 (36)
2	12 (21)
3	5 (9)
Radiographic bone loss	
Slight	4 (7)
Moderate	15 (27)
Severe	22 (39)
Absence of PDL space (radiographic)	39 (70)
Pain	33 (59)
Extrusion	11 (20)
Past-periodontitis	38 (67)
Current-periodontitis	43 (77)
Periodontal treatment-related abscess	2 (3.5)
Trauma-related abscess	2 (3.5)

BOP, bleeding on probing; PD, probing depth, PDL (Periodontal Ligament).

**Table 3:** Percentage of cultivable bacterial species in periodontal abscesses.

Microorganism	Frequency of Detection (*n* = 56), % (*n*)	Periodontal Abscess (*n* = 56), % ±SD
*Prevotella intermedia/nigrescens*	64 (36)	10.35 ± 15.40
*Fusobacterium* spp.	73 (41)	4.45 ± 3.430
*Porphyromonas gingivalis*	46 (26)	4.34 ± 4.90
Gram-negative enteric rods	20 (11)	3.30 ± 8.05
*Tannerella forsythia*	13 (7)	0.94 ± 1.63
*Campylobacter* spp.	11 (6)	0.45 ± 1.40
*Actinobacillus actinomycetemcomitans*	25 (14)	0.11 ± 0.23

**Table 4:** Susceptibility of selected bacterial species isolated from periodontal abscess to various antibacterial agents.

Antimicrobial	*Actinobacillus actinomycetemcomitans* (*n* = 6)		*Porphyromonas gingivalis* (*n* = 10)		*Prevotellaintermedia*/*nigrescens* (*n* = 10)	
	Susceptible	Resistant	Susceptible	Resistant	Susceptible	Resistant
Tetracycline	5	1	10	0	10	0
Metronidazole	2	4	9	1	10	0
Azithromycin	6	0	10	0	10	0
Amoxicillin	4	2	10	0	8	2
